# Neuroimaging of Natalizumab Complications in Multiple Sclerosis: PML and Other Associated Entities

**DOI:** 10.1155/2015/809252

**Published:** 2015-09-21

**Authors:** Justin M. Honce, Lidia Nagae, Eric Nyberg

**Affiliations:** Division of Neuroradiology, University of Colorado School of Medicine, 12700 E 19th Avenue Mail Stop C278, Aurora, CO 80045, USA

## Abstract

Natalizumab (Tysabri) is a monoclonal antibody (*α*4 integrin antagonist) approved for treatment of multiple sclerosis, both for patients who fail therapy with other disease modifying agents and for patients with aggressive disease. Natalizumab is highly effective, resulting in significant decreases in rates of both relapse and disability accumulation, as well as marked decrease in MRI evidence of disease activity. As such, utilization of natalizumab is increasing, and the presentation of its associated complications is increasing accordingly. This review focuses on the clinical and neuroimaging features of the major complications associated with natalizumab therapy, focusing on the rare but devastating progressive multifocal leukoencephalopathy (PML). Associated entities including PML associated immune reconstitution inflammatory syndrome (PML-IRIS) and the emerging phenomenon of rebound of MS disease activity after natalizumab discontinuation are also discussed. Early recognition of neuroimaging features associated with these processes is critical in order to facilitate prompt diagnosis, treatment, and/or modification of therapies to improve patient outcomes.

## 1. Introduction

**Figure 1:**
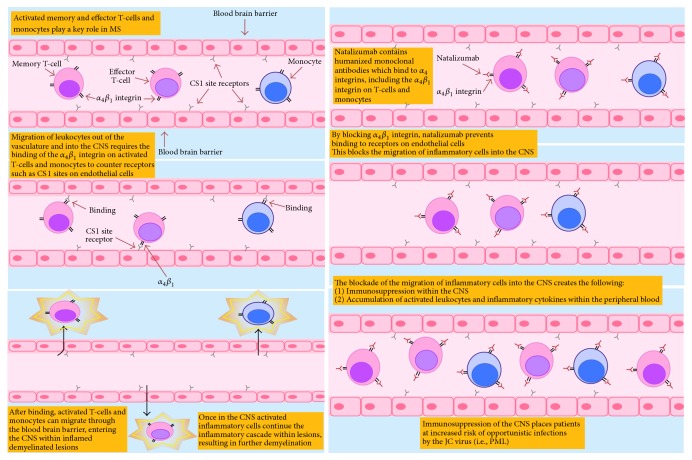
Pathophysiology of MS and mechanism of action of natalizumab.

Natalizumab is a monoclonal antibody directed against the *α*4*β*1 and *α*4*β*7 integrins, approved for therapy in relapsing remitting multiple sclerosis [1]. The drug prevents inflammatory cells from binding to cerebrovascular endothelial cells, thereby preventing them from crossing the blood brain barrier and entering the brain [1, 2], resulting in profound immunosuppression within the CNS (Figure 1). This blockade is a highly effective therapy for multiple sclerosis, with placebo controlled studies demonstrating up to a 68% reduction in clinical relapse rates, 42% reduced risk of sustained progressive disability, 92% fewer gadolinium enhancing lesions, and an 83% decrease in the accumulation of new or enlarging T2 hyperintense white matter lesions [3–5]. Up to 37% of patients appear completely free of clinical and radiological disease activity while on therapy over 2 years [6]. Given its clinical efficacy, utilization of natalizumab has rapidly increased, with approximately 134,800 MS patients receiving or having received the drug as of March 2015 (https://medinfo.biogen.com/, accessed June 2015). With increasing utilization of natalizumab, radiologists and neurologists are more likely to encounter its complications in clinical practice, and prompt recognition of these complications is critical for optimal management.

The overall incidence of adverse events associated with natalizumab is low. Infusion and allergic reactions have been reported in small groups of patients but are managed efficiently with corticosteroids [7]. Hepatic injuries have been reported to occur after the first infusion, though they are not common [8, 9]. Several cases of melanoma have been reported in patients on natalizumab [10–12], but incidence appears similar between placebo and natalizumab and there is insufficient evidence to support a definitive link to natalizumab [11, 13, 14]. There have been 6 reported cases of CNS lymphoma in patients treated with natalizumab [15–19]. However, two of these patients may have had preexisting lymphoma and at least one was negative for EBV, suggesting that these lymphomas may not have been caused by natalizumab therapy, though potentiation of progression is not excluded [16, 20].

The primary complication of natalizumab therapy is progressive multifocal leukoencephalopathy (PML). Rapid drug removal, usually by plasma exchange (PLEX), may contribute to improved patient survival, but early diagnosis is crucial [21–23]. Unfortunately, despite successful management of PML, PML associated immune reconstitution inflammatory syndrome (PML-IRIS) may occur resulting in a paradoxical worsening of symptoms. The IRIS phenomenon is not limited to PML treatment and is emerging in a subset of patients upon cessation of natalizumab therapy for other reasons who experience exuberant rebound of MS disease activity after natalizumab discontinuation. These three phenomena, PML, PML-IRIS, and natalizumab rebound, each have significant negative effects on patient morbidity and mortality and are the main focus of this review. Early recognition of the spectrum of clinical and imaging findings is crucial in order to limit their devastating impact.

## 2. PML: Background

PML is an opportunistic infection of the brain caused by the JC virus, affecting severely immunosuppressed patients with impaired T-lymphocyte responses [24]. Early investigations of PML-infected brains demonstrated that the JC virus predominantly infects myelin-producing oligodendrocytes resulting in severe irreversible demyelination [25, 26]. While oligodendrocytes are the primary site of CNS infection, the virus has also been detectable in astrocytes [27] and cell loss in the granule layer of the cerebellum and neuronal infections have been reported [28–30]. JCV viral infection is widespread, with serum antibodies against JC virus detectable in as much as 80% of the population [31]. PML may result from reactivation of latent JC virus infection in the brain [26] or spread from peripheral reservoirs in the kidneys [32] and bone marrow [33] to the brain during immunosuppression.

PML was originally reported in rare association with lymphoma. As HIV emerged, the incidence of PML also increased, with up to 5% of AIDS patients contracting the disease [34]. PML has also been reported in patients with rheumatic diseases such as lupus, those receiving organ transplants and patients taking immunosuppressive therapies such as alkylating agents, purine analogues, and monoclonal antibodies [35]. Besides natalizumab, PML has recently been reported in two MS patients treated with fingolimod [36] and one patient treated with tecfidera [37] without prior exposure to other immunosuppressants. PML has also been reported in 3 patients receiving dimethyl fumarate (DMF), both with and without lymphopenia [38–40]. While these patients were being treated for psoriasis, not MS, DMF is also used in the treatment of MS, necessitating vigilant monitoring for PML in this patient group as well.

## 3. Natalizumab and PML

Three cases of PML were reported in patients being treated with natalizumab, soon after introduction of the drug in the US market in 2004. Two of the cases occurred in MS patients and one in a patient being treated for Crohn's disease [41–43]. In February 2005 clinical trials and commercial dosing were voluntarily suspended due to these three cases. In July 2006, however, the drug was reintroduced, with additional precautions, when studies demonstrated no new cases of PML in previously treated patients [44]. These new precautions included mandatory risk mitigation and restriction to RRMS patients with high disease activity. Despite these new precautions, three additional cases of natalizumab associated PML were identified by 2008 and, since then, the number of reported PML cases has steadily risen, with 42 cases by March 2010, 372 cases by June 2013, and 566 confirmed cases as of June 2015 (https://medinfo.biogen.com/, accessed July 2015). The actual number of PML infections may be even higher as PML is frequently underdiagnosed as many PML cases may be missed or misclassified. The global overall risk of PML for patients on natalizumab therapy is estimated to be 3.96 per 1000 patients (95% CI 3.64–4.30) (https://medinfo.biogen.com/, accessed July 2015). The presence of JC virus antibodies in the blood of MS patients is a risk factor for PML development, stratified by the JVC antibody index: an index >0.4 indicates positivity and <0.2 indicates negativity, while an index between 0.2 and 0.4 denotes an indeterminate response [45]. The estimated incidence for MS patients who test negative (index < 0.2) for JC virus antibody is extremely low, less than 0.11 per 1000 (95% CI 0.00–0.59), and reaches 5.55 per 1000 (95% CI 5.34–6.42) in those testing positive (index > 0.4) without other risk factors. The risk of PML increases with longer treatment duration, peaking at 24 months with a risk of 6.11 per 1000 (95% CI 5.35–6.47). A prior history of immunosuppressant use [35] further increases risk, reaching a maximum incidence of 13 per 1000 in JCV-Ab + patients on natalizumab greater than 49 months and with prior immunosuppressive therapy exposure (https://medinfo.biogen.com/, accessed July 2015).

## 4. Natalizumab and PML: Prognosis

The mortality rate in natalizumab associated PML is approximately 22% [46]. This is considerably lower than the more common HIV-AIDS associated form, which has been reported in up to 40–50% in the HAART era [23, 46]. Those who survive, however, usually suffer from significant disability with 90% of patients having moderate or severe disability per Karnofsky Performance Scale (KPS) scores 6 months after diagnosis (https://medinfo.biogen.com/, accessed July 2015). Mortality rates are higher in patients who are older and have poorer baseline function related to the severity of MS and in those in whom diagnosis was delayed [23]. The presence or absence of symptoms at the time of diagnosis appears to be an important prognostic factor. Asymptomatic patients demonstrate significantly lower mean Expanded Disability Status Scale (EDSS) scores, higher mean KPS scores, and improved survival compared with symptomatic patients [47].

## 5. Natalizumab and PML: Clinical Features

The clinical presentation of PML is heterogeneous and may include focal and nonfocal neurologic deficits affecting neurobehavioral, motor, language, and visual functions [21, 48]. Cognitive deficits are not surprisingly the most common, given the widely distributed nature of cognitive function throughout the brain and the already compromised neural condition of MS patients. Although quite rare, brain stem involvement causes the most severe symptoms. While PML can be detected in patients who are asymptomatic [47, 49], the earliest symptoms attributable to PML are usually nonspecific and subtle and may be misinterpreted as exacerbations of multiple sclerosis, related to depression or may be missed entirely [50]. Symptoms in the mid and later stages of the disease can be mistakenly diagnosed as stroke or seizure disorders, and seizures have been reported in up to 20% of patients with PML [51]. Therefore, PML should be considered in the differential diagnosis of any MS patient taking natalizumab presenting with new neurologic symptoms. As the name implies, the disease is progressive and symptoms worsen over time. Moreover, if this entity is not recognized and diagnosis is delayed, symptom progression may accelerate.

## 6. PML: Surveillance and Diagnosis

Confident diagnosis of PML is achieved through a combination of clinical features, characteristic imaging findings, laboratory testing, and histopathology as outlined in the recently proposed case definition for natalizumab associated PML and in the AAN consensus statement on PML diagnostic criteria [52, 53]. These guidelines emphasize that the highest level of diagnostic certainty requires histopathologic confirmation but that the presence of clinical and/or imaging findings in combination with JCV DNA in the CSF is also diagnostic. These criteria not only provide some certainty to the diagnosis, but also serve as a guideline on what further testing could be obtained to achieve a more conclusive diagnosis.

Currently there is no consensus on how frequently surveillance MRI should be performed to monitor for PML, with some suggesting scanning up to every 3-4 months [54]. When PML is suspected clinically, timely MRI imaging and cerebrospinal fluid sampling with polymerase chain reaction (PCR) testing for JC virus DNA should be performed to confirm diagnosis. PCR for JCV DNA in the CSF has a reported sensitivity of 80% and specificity of 90% [55].

Negative CSF PCR for JCV does not exclude PML as viral loads can be very low (<100 copies/mL), and most commercial tests are only able to detect JCV DNA in excess of 200 copies/mL [21, 56]. Patients with repeatedly negative CSF JCV PCR can nevertheless demonstrate MR imaging suggestive of PML [22, 44, 52, 57]. A recently proposed test to help confirm the diagnosis of PML in such cases relies on the elevation of anti-JCV IgG antibodies in the CSF and the calculation of the CSF JCV antibody index: an index of >1.5 was 100% specific and 57% sensitive for the diagnosis of PML in these cases [56]. In difficult cases where the clinical, radiologic, or laboratory findings are inconclusive, brain biopsy can also be performed [53].

## 7. PML: Imaging Features

CT imaging abnormalities in patients with PML have been described, generally demonstrating multiple areas of low attenuation with scalloped borders in the peripheral and subcortical white matter, with these areas coalescing as the disease progresses [58].

**Figure 2:**
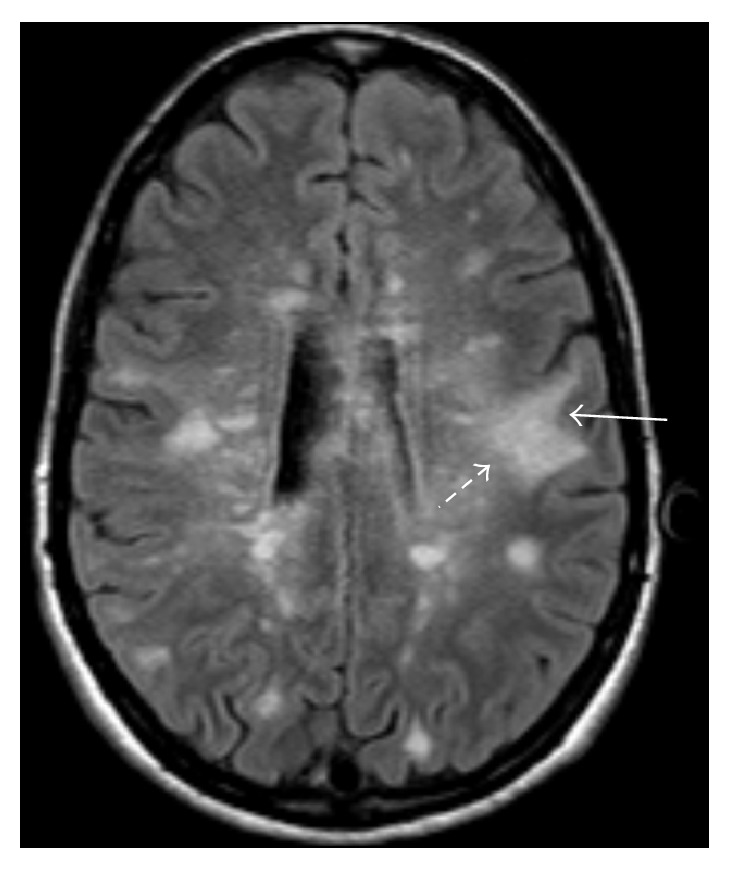
25-year-old woman with RRMS who developed worsening symptoms including weakness and inability to ambulate after beginning natalizumab. MRI FLAIR image demonstrates classic appearance of PML including a sharply demarcated peripheral border along the subcortical U-fibers (arrow) and a hazy, ill-defined central border (dashed arrow).

**Figure 3:**
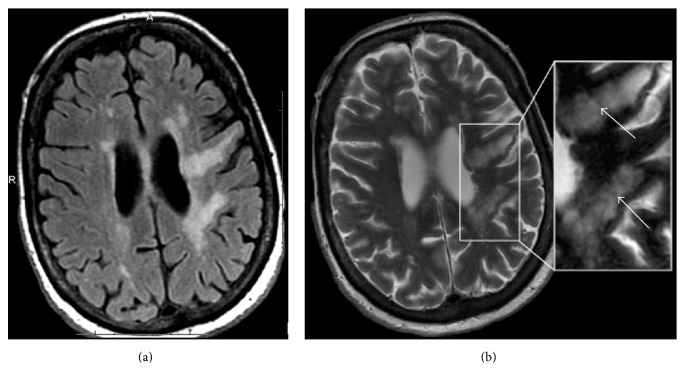
63-year-old woman with MS presenting with profound neurologic deterioration. (a) FLAIR image demonstrates characteristic PML lesions involving the subcortical U-fibers which also extent centrally to the periventricular surface. (b) T2-weighted image on the same patient demonstrates “granular” or “microcystic” foci (arrows).

In the current era, MRI has supplanted CT as the modality of choice for the diagnosis of PML. PML characteristically presents as one or more areas of T2/FLAIR hyperintensity in the white matter in a peripheral, bilateral, but asymmetric distribution. Lesions vary in shape and size, growing larger and becoming confluent as the disease progresses. Lesions classically involve the subcortical U-fibers in nearly all cases [59]. This subcortical involvement leads to a sharp border between the superficial aspect of the lesion and the overlying cortex, while the deeper border remains ill defined (Figure 2). Involvement of the overlying cortex, while originally thought to be rare, has been increasingly reported [59–63]. As the disease is usually peripheral, the periventricular white matter is typically spared [48]; however, the periventricular location does not preclude the possibility of PML (Figure 3(a)).

On T1 weighted imaging lesions become increasingly hypointense, as irreversible white matter destruction occurs [44, 48, 59]. T2-weighted imaging may demonstrate a “microcyst” or “granular” pattern, especially in larger lesions [48, 59]. This finding has been suggested to represent small areas of demyelination which occur in the immediate vicinity of infected oligodendrocytes or early immune response within perivascular spaces [59] (Figure 3(b)).

**Figure 4:**
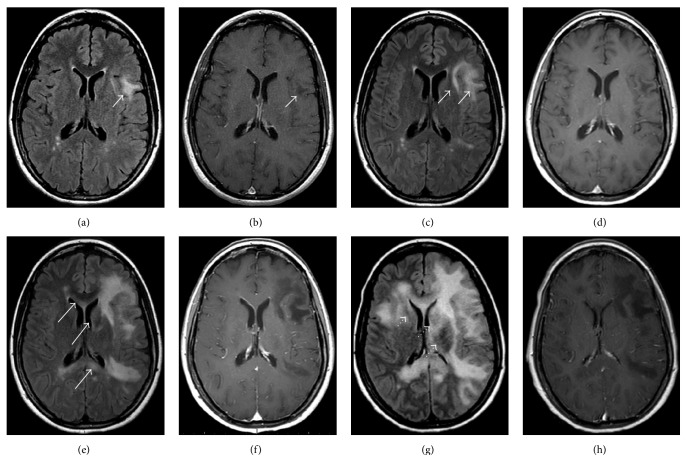
32-year-old woman with RRMS and no new neurologic symptoms developed MR findings on FLAIR images (a) without enhancement (b) consistent with PML (arrows) after having been on natilizumab for approximately 4.5 years. The drug was discontinued and she received PLEX and steroids. Follow-up imaging was obtained at (c-d) one month, (e-f) three months, and (g-h) four months later demonstrating progressive PML lesions, which corresponded to progressive clinical neurological decline. FLAIR (c) and postcontrast (d) images obtained at one month demonstrate disease progression without enhancement. Images obtained at three months show progressive disease (e-f) without enhancement to suggest active MS or IRIS. Like other aggressive infiltrating white matter lesions PML can cross the corpus callosum (arrows). Note also that there is now involvement of the left caudate (dotted arrow). The final images obtained four months after presentation (g-h) demonstrate swelling and compression of the gyri. There is now marked involvement of the deep gray matter structures (dotted arrows) which occurs in up to one-third of cases. However, note that despite involvement of almost the entire hemisphere, the lateral ventricle remains only mildly compressed and there is no midline shift, as would be expected with other lesions of this size, and no enhancement has developed.

The regular use of MRI imaging in MS patients may be able to detect the disease very early before the patients become symptomatic [49, 64–67]. Imaging at these stages typically shows hazy, typically hyperintense T2 signal in the juxtacortical white matter and usually involves the U-fibers, most commonly in a single lobe. The lesions may not be as focal as a typical MS relapse and generally do not enhance while the patient is asymptomatic (Figures 4(a) and 4(b)). Gray matter involvement may be present in up to 83%. 60% of asymptomatic patients have lesions which show high signal intensity on DWI [67].

In the supratentorial brain the parietal, occipital, and frontal lobes are the most frequently involved. Like other aggressive infiltrating lesions, PML can infiltrate the corpus callosum, though isolated corpus callosal involvement is rare (Figures 4(d) and 4(f)). Deep gray matter involvement has been reported in conjunction with white matter lesions in up to 5–31% of patients in some series [48, 68, 69]. The thalami are more commonly involved than the basal ganglia. Cortical involvement is increasingly recognized as well. In the early stages of the disease there is typically no mass effect; however, as lesions progress mild mass effect can develop. The degree of mass effect appears mild compared to the extent of disease (Figures 4(d) and 4(e)).

**Figure 5:**
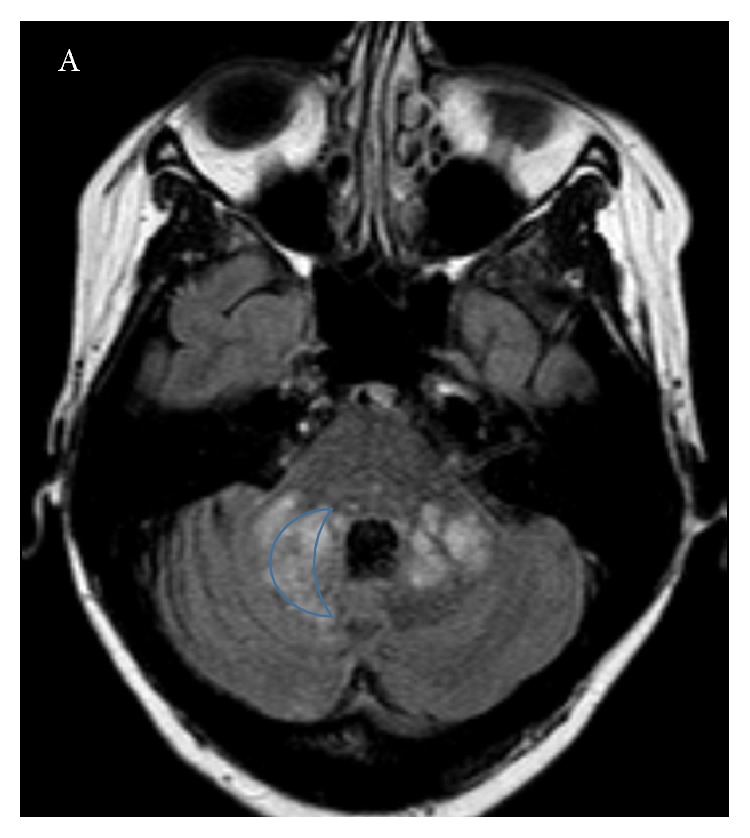
45-year-old woman with a 10-year history of RRMS was started on natalizumab. She did well for six years and then developed gait abnormality and fatigue. MR imaging demonstrates large lesions in the cerebellar peduncles demonstrating a “crescent” shape.

Posterior fossa involvement is frequently reported, most commonly involving the cerebellum and middle cerebellar peduncles, although the brainstem can also be involved [70]. “Crescent” shaped lesions involving middle cerebellar peduncles and adjacent cerebellar and/or pontine white matter may be specific to PML, rather than MS, as they have so far only been reported in PML patients (Figure 5) [48, 70–73]. The optic nerve and spinal cord are spared. Hemorrhage is a rare finding that has been reported on occasion in the literature for HIV patients taking natalizumab [74] and is rarely seen in PML [41].

The incidence of contrast enhancement at initial diagnostic imaging in natalizumab associated PML is higher than that in HIV populations, with up to 43% of natalizumab symptomatic PML cases reporting contrast enhancement at diagnosis [21], compared with 15% in HIV populations [25]. The pattern of enhancement is variable and may be patchy, linear, nodular, or peripheral and in some cases demonstrates a perivascular pattern [75]. Enhancement at time of diagnosis is correlated with decreased survival and greater clinical disability than those that do not enhance [76]. Enhancement suggests that in this subset of patients natalizumab associated PML involves an inflammatory response to the JCV infection, despite the immunosuppression provided by the drug. Therefore, new enhancing lesions on MRI in patients on natalizumab should not necessarily be assumed to be MS relapse [21].

## 8. Diffusion Imaging

**Figure 6:**
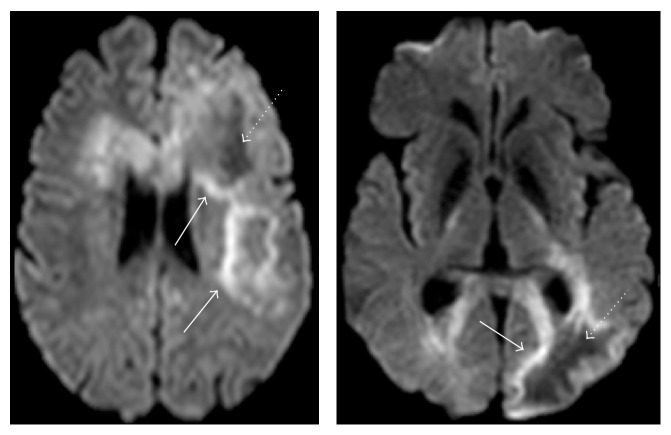
Diffusion weighted images in a patient with large PML lesions demonstrate peripheral restricted diffusion where the lesion is active (arrows) and central facilitated diffusion where the lesion is more quiescent (dotted arrows).

**Figure 7:**
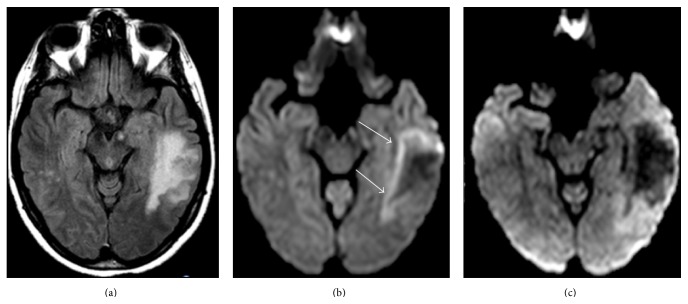
(a) T2 FLAIR and (b) DWI images demonstrate a large PML lesion. (b) DWI demonstrates cytotoxic edema along the advancing edge of the lesion (arrows) surrounding the quiescent center. (c) Repeat MR DWI image following PLEX demonstrates absence of the hyperintense rim suggesting that disease progression has resolved.

Conventional T2-weighted and T2 FLAIR imaging is sensitive to increased water in brain tissue. However, T2 does not differentiate between cytotoxic processes resulting in intracellular edema, for example, in the setting of cell injury or death, and vasogenic interstitial edema. Diffusion weighted MR imaging (DWI), however, is highly sensitive to the restriction of Brownian diffusion of water molecules which occurs in the setting of cellular injury and cytotoxic edema [77]. The DWI appearance of PML lesions varies depending on the stage of the disease. Early in the course of the disease when lesions are relatively small, infected oligodendrocytes swell and die, resulting in high signal on DWI [78]. As the lesions enlarge the signal on DWI remains high within the peripheral as new oligodendrocytes become infected [78–80] (Figure 6). As treatment is initiated and the lesions become quiescent, the rim loses its DWI hyperintensity (Figure 7). Over time, the more typical appearance of low signal on DWI develops due to later phases of tissue destruction and compromise of the blood brain barrier resulting in relatively free diffusion of water within the damaged tissue [81]. Pathologic correlation has suggested that this lesion core corresponds to areas of dead and shrunken oligodendrocytes, bizarre astrocytes, and numerous macrophages and indicates irreversibly destroyed white matter [78]. Since the T1 and T2 signal changes associated with PML are in general irreversible, DWI is an essential tool for monitoring disease progression and treatment response [81–85].

Diffusion tensor imaging (DTI) has emerged as a useful imaging modality for detection of microstructural changes in the white matter, including myelination, and assessing white matter integrity [83]. In PML fractional anisotropy (FA) values are reduced, compatible with myelin injury. These changes occur very early in the disease and may detect PML before conventional imaging shows abnormalities [79]. As white matter injury progresses ADC values rise [81, 86], compatible with more irreversible damage.

## 9. MR Spectroscopy

MRS in PML lesions typically demonstrates lower levels of N-acetylaspartate, elevated levels of choline (Cho), and variable myoinositol levels. Spectra differ somewhat between the center and periphery of the lesion and depend on the phase of disease. In general, the periphery of the lesion demonstrates greater increases in Cho and less notable decreases in NAA than in the center of the lesion. This corresponds to more active demyelination peripherally and more advanced neuronal destruction centrally [87–89]. Unfortunately MRS findings in PML are nonspecific, with similar spectra seen in multiple other types of CNS lesions, including malignancies and MS plaques.

## 10. PML: Differential Diagnoses

It is important to consider the differential diagnosis for new MRI findings in MS patients receiving natalizumab as this will affect treatment decisions. JCV infection is the most common opportunistic infection, but other viral CNS infections, including varicella zoster myelitis and herpes simplex encephalitis, have also been reported in natalizumab patients [90]. Varicella-zoster myelitis may demonstrate T2 hyperintense signal and enhancement in the spinal cord [91], differentiating it from PML. Herpes zoster encephalitis presents as rapidly progressive cortical and subcortical T2 hyperintensity, swelling, and occasional enhancement involving the temporal lobes +/− other limbic regions [92]. Various bacterial and fungal infections may also occur.

The most important differential consideration is whether new lesions are related to multiple sclerosis relapse. New MS lesions tend to be small, focal, and well delineated, favoring the periventricular and juxtacortical white matter and are typically round or ovoid in shape [48, 69]. MS lesions may enhance homogenously or peripherally, whereas PML generally does not. MS lesions generally only restrict diffusion in the hyperacute phase (<1 week) [93].

Tumefactive demyelinating can be misinterpreted for PML lesions as they demonstrate large areas of T2 hyperintensity and T1 hypointensity; however, mass effect is usually greater in tumefactive lesions [94], and T1 hypointensity improves over time in tumefactive lesions due to remyelination [94–96], while this does not occur in PML. Acute disseminated encephalomyelitis (ADEM) can appear similar to PML with large areas of T2 signal abnormality in the white matter and deep gray structures with minimal enhancement and variable mass effect [97]. Posterior reversible encephalopathy syndrome (PRES) can appear superficially similar to PML on initial examination, but lesions tend to be more symmetric than those in PML and predominantly involve the posterior aspects of the brain. Finally, PRES lesions typically resolve with treatment of the inciting etiology [98].

## 11. PML: Treatment

The goal of treatment for natalizumab associated PML is the restoration of immune function by expedient removal of the drug. This is typically achieved with plasma exchange (PLEX) or immunoadsorption (IA), which clears natalizumab from the *α*4*β*1 receptors. Three to five PLEX sessions may be required over the course of 2 weeks [99]; however, more or fewer sessions may be needed and serum natalizumab level monitoring during PLEX may be of benefit [100].

## 12. PML-IRIS

**Figure 8:**
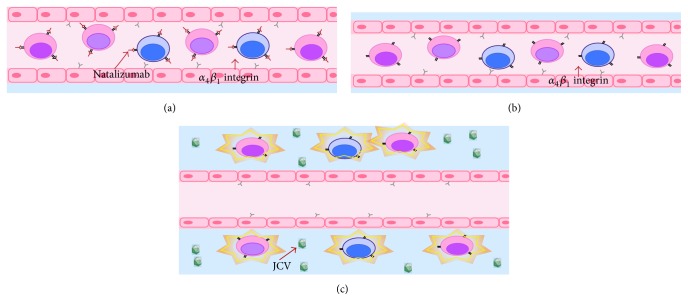
Pathophysiology of PML-IRIS: (a) natalizumab blocks the *α*4*β*1 integrin, preventing lymphocyte tracking into the CNS. (b) To treat PML natalizumab must be rapidly cleared from the blood, often through PLEX. (c) With natalizumab effectively cleared from *α*4*β*1 integrin receptors the lymphocytes reenter the CNS to attack the PML virus. The response is often overwhelming, possibly exacerbating IRIS and leading to further destruction of brain tissue.

Immune Reconstitution Inflammatory Syndrome (IRIS) is a phenomenon originally reported in AIDS patients who were prescribed highly active antiretroviral therapy and subsequently experienced a paradoxical clinical deterioration [101]. In PML patients treated with PLEX, once clearance of natalizumab has been achieved, many patients will experience rapid worsening of neurologic symptoms. This is thought to be due to an exuberant immune response to viral antigens resulting in inflammation mediated damage to infected and noninfected neuronal and glial tissue. Given the strong association between natalizumab associated PML and IRIS, the combined term PML-IRIS is used to describe this phenomenon (Figure 8) [66].

PML-IRIS may occur following discontinuation of natalizumab in the absence of PLEX; however, it tends to occur later, usually approximately 90 days after last dose, reflecting the longer time necessary to clear the drug [21, 102]. The clinical impact of the PML-IRIS phenomenon should not be understated, as it results in substantially worsened EDSS scores and up to 30% mortality [23]. The most common therapy for PML-IRIS is high dose corticosteroids in an attempt to control the deleterious effects of the exuberant inflammatory cascade [35, 66, 76].

## 13. PML-IRIS: Imaging Features

**Figure 9:**
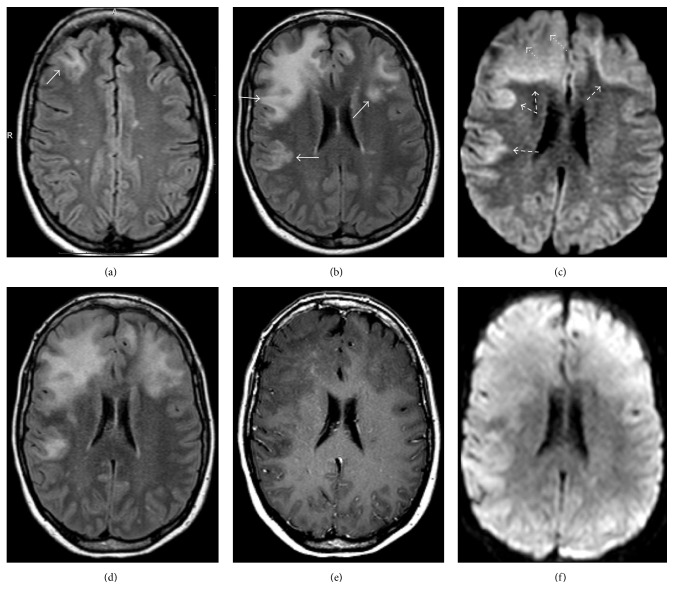
39-year-old woman with RRMS treated with natalizumab developed worsening symptoms. (a) MRI was performed at an outside institution demonstrating a new subcortical lesion in the right frontal lobe (arrow). She was treated for MS exacerbation. The patient presented to our institution approximately 3 months later with progressive symptoms and functional decline. (b) Repeat MRI shows increase in size of the right frontal lesion with characteristic bilateral, asymmetric distribution of lesions involving the subcortical U-fibers (arrows). (c) DWI shows a bright rim of signal along the advancing edge of the lesion (dashed arrows) with darker signal more anteriorly where the lesion is no longer active (dotted arrows). At this time there was no contrast enhancement (not shown). The patient was diagnosed with PML and PLEX was performed. Approximately one month later the patient experienced functional decline. Repeat MRI shows expansion of the (d) FLAIR lesions with increased swelling and mass effect and (e) the development of patchy central enhancement consistent with PML-IRIS. Note that the DWI image (f) no longer demonstrates an advancing edge of restricted diffusion.

Given the substantial morbidity and mortality associated with PML-IRIS, prompt recognition of the phenomenon is crucial for rapid initiation of supportive therapies. Progression of the typical imaging findings of PML and evidence of active inflammation following clearance of natalizumab from the patient's system are the hallmarks of PML-IRIS. Existing PML lesions may increase in size and may coalesce as more white matter becomes involved. This is accompanied by increasing edema, cerebral swelling, and mass effect, which are not typical of PML. Contrast enhancement develops or increases and exhibits variable patterns, including patchy, punctate, irregular and hazy, ill-defined enhancement patterns (Figure 9) [59]. These inflammatory MR findings will progress then regress over time. Lesions remain T2/FLAIR hyperintense after cessation of the active inflammatory response. Typically T1 hypointensity increases indicating irreversible white matter damage. Long term retrograde neuronal degeneration results in atrophy of the overlying cortex.

## 14. Natalizumab Rebound

**Figure 10:**
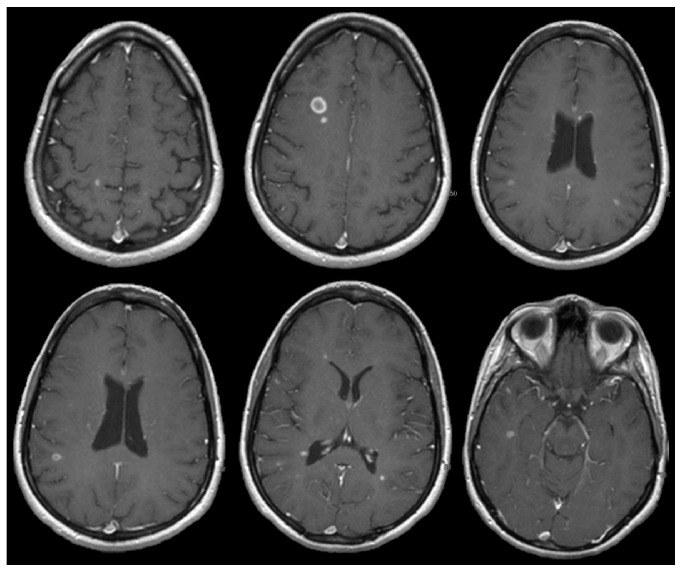
27-year-old female with a typical relapse severity prior to natalizumab of 0–2 enhancing lesions on MRI. The patient was treated with natalizumab for 30 months without clinical or radiologic relapse but ceased natalizumab due to JCV Ab conversion. 3.3 months after cessation, patient relapsed and imaging demonstrated rebound with >14 enhancing lesions.

Patients may find it necessary to discontinue natalizumab therapy for a variety of reasons including the fear of contracting PML after long term usage, JC virus seroconversion, disease progression despite treatment, pregnancy or the intention to become pregnant, antibodies to natalizumab, or allergy [103]. Multiple small studies have demonstrated an unusually robust inflammatory response greater than a patient's typical relapse severity before starting natalizumab therapy (i.e., rebound) on MR imaging performed within approximately three months following discontinuation of the drug [104–112]. The picture is not definitive, however, as phase III clinical trial data shows renewed disease progression at an expected pre-natalizumab level [113]; however, other trials demonstrate disease severity greater than what had been previously experienced. This is referred to as the “rebound phenomenon” [114, 115] and may occur in 10–40% of patients after discontinuation of natalizumab [108, 109, 116]. Rebound may be more frequent in those patients with a lower pretreatment level of disease activity and in those patients in whom the gap between discontinuation of natalizumab and initiation of another subsequent therapy is delayed [103, 117]. Therefore, clinicians need to be vigilant when monitoring patients after cessation of natalizumab, as the disease may become more aggressive during this period, resulting in a more profound relapse than would otherwise be expected. On MR imaging the appearance of the rebound phenomenon appears as new enhancing and/or nonenhancing lesions. The number of new or enhancing lesions may be greater than in a typical relapse and can be quite severe [108–110] (Figure 10). Development of enhancement at the margins of old lesions has also been reported [109].

## 15. Conclusion

Given the widespread and increasing use of natalizumab for the treatment of RRMS it is crucial for the neurologist and neuroradiologist to understand and recognize the common major complications of this treatment. These primarily include PML, PML-IRIS, and natalizumab rebound. The imaging presentation of PML is typified by bilateral but asymmetric areas of abnormal T2 signal in the peripheral subcortical white matter, with or without enhancement. Diffusion weighted imaging is of particular value in the evaluation of patients suspected of PML, as peripheral hyperintensity and central hypointensity on DWI images are classic. DWI may have utility in differentiating early PML from MS relapse and may be used to monitor patients treated for PML. Although not yet in widespread clinical use, DTI may be able to detect PML earlier than conventional imaging, before it is clinically manifest. PML is treated by rapid clearance of natalizumab from the patient with PLEX. Clearance of natalizumab, with or without PLEX, often results in PML-IRIS, which is typified by progression of the typical imaging findings of PML and evidence of new contrast enhancement and swelling. Finally, the phenomenon of natalizumab rebound after drug discontinuation, while controversial, may be a distinct entity in a subset of patients. This is essentially an aggressive relapse and may demonstrate more numerous enhancing lesions as well as enhancement of the margins of old lesions.

## References

[1] Lutterotti A., Martin R. (2008). Getting specific: monoclonal antibodies in multiple sclerosis. *The Lancet Neurology*.

[2] Berger J. R. (2006). Natalizumab. *Drugs of Today*.

[3] Polman C. H., O'Connor P. W., Havrdova E. (2006). A randomized, placebo-controlled trial of natalizumab for relapsing multiple sclerosis. *The New England Journal of Medicine*.

[4] Miller D. H., Soon D., Fernando K. T. (2007). MRI outcomes in a placebo-controlled trial of natalizumab in relapsing MS. *Neurology*.

[5] Rudick R. A., Stuart W. H., Calabresi P. A. (2006). Natalizumab plus interferon beta-1a for relapsing multiple sclerosis. *The New England Journal of Medicine*.

[6] Havrdova E., Galetta S., Hutchinson M. (2009). Effect of natalizumab on clinical and radiological disease activity in multiple sclerosis: a retrospective analysis of the natalizumab safety and efficacy in relapsing-remitting multiple sclerosis (AFFIRM) study. *The Lancet Neurology*.

[7] Hellwig K., Schimrigk S., Fischer M. (2008). Allergic and nonallergic delayed infusion reactions during natalizumab therapy. *Archives of Neurology*.

[8] Lisotti A., Azzaroli F., Brillanti S., Mazzella G. (2012). Severe acute autoimmune hepatitis after natalizumab treatment. *Digestive and Liver Disease*.

[9] Bezabeh S., Flowers C. M., Kortepeter C., Avigan M. (2010). Clinically significant liver injury in patients treated with natalizumab. *Alimentary Pharmacology and Therapeutics*.

[10] Mullen J. T., Vartanian T. K., Atkins M. B. (2008). Melanoma complicating treatment with natalizumab for multiple sclerosis. *The New England Journal of Medicine*.

[11] Bergamaschi R., Montomoli C. (2009). Melanoma in multiple sclerosis treated with natalizumab: causal association or coincidence?. *Multiple Sclerosis*.

[12] Ismail A., Kemp J., Sharrack B. (2009). Melanoma complicating treatment with Natalizumab (Tysabri) for multiple sclerosis. *Journal of Neurology*.

[13] Laroni A., Bedognetti M., Uccelli A., Capello E., Mancardi G. L. (2011). Association of melanoma and natalizumab therapy in the Italian MS population: a second case report. *Neurological Sciences*.

[14] Panzara M. A., Bozic C., Sandrock A. W. (2008). More on melanoma with transdifferentiation. *The New England Journal of Medicine*.

[15] Schweikert A., Kremer M., Ringel F. (2009). Primary central nervous system lymphoma in a patient treated with natalizumab. *Annals of Neurology*.

[16] Bozic C., LaGuette J., Panzara M. A., Sandrock A. W. (2009). Natalizumab and central nervous system lymphoma: no clear association. *Annals of Neurology*.

[17] Phan-Ba R., Bisig B., Deprez M. (2011). Primary central nervous system lymphoma in a patient treated with natalizumab. *Annals of Neurology*.

[18] Matzke M., Schreiber S., Elolf E. (2012). Natalizumab-associated central nervous system lymphoma?—another patient. *Multiple Sclerosis*.

[19] Na A., Hall N., Kavar B., King J. (2014). Central nervous system lymphoma associated with natalizumab. *Journal of Clinical Neuroscience*.

[20] Goebels N., Kappos L. (2009). Another complication of natalizumab treatment? Taking the challenge. *Annals of Neurology*.

[21] Clifford D. B., DeLuca A., Simpson D. M., Arendt G., Giovannoni G., Nath A. (2010). Natalizumab-associated progressive multifocal leukoencephalopathy in patients with multiple sclerosis: lessons from 28 cases. *The Lancet Neurology*.

[22] Dahlhaus S., Hoepner R., Chan A. (2013). Disease course and outcome of 15 monocentrically treated natalizumab-associated progressive multifocal leukoencephalopathy patients. *Journal of Neurology, Neurosurgery and Psychiatry*.

[23] Vermersch P., Kappos L., Gold R. (2011). Clinical outcomes of natalizumab-associated progressive multifocal leukoencephalopathy. *Neurology*.

[24] Tan C. S., Koralnik I. J. (2010). Progressive multifocal leukoencephalopathy and other disorders caused by JC virus: clinical features and pathogenesis. *The Lancet Neurology*.

[25] Berger J. R., Major E. O. (1999). Progressive multifocal leukoencephalopathy. *Seminars in Neurology*.

[26] Sabath B. F., Major E. O. (2002). Traffic of JC virus from sites of initial infection to the brain: the path to progressive multifocal leukoencephalopathy. *The Journal of Infectious Diseases*.

[27] Ledoux S., Libman I., Robert F., Just N. (1989). Progressive multifocal leukoencephalopathy with gray matter involvement. *Canadian Journal of Neurological Sciences*.

[28] Richardson E. P., Webster H. D. (1983). Progressive multifocal leukoencephalopathy: its pathological features. *Progress in Clinical and Biological Research*.

[29] Du Pasquier R. A., Corey S., Margolin D. H. (2003). Productive infection of cerebellar granule cell neurons by JC virus in an HIV+ individual. *Neurology*.

[30] Wüthrich C., Dang X., Westmoreland S. (2009). Fulminant JC virus encephalopathy with productive infection of cortical pyramidal neurons. *Annals of Neurology*.

[31] Tornatore C., Amemiya K., Atwood W., Conant K., Major E. O., Berger J. (1994). JC virus: current concepts and controversies in the molecular virology and pathogenesis of progressive multifocal leucoencephalopathy. *Reviews in Medical Virology*.

[32] Chesters P. M., Heritage J., McCance D. J. (1983). Persistence of DNA sequences of BK virus and JC virus in normal human tissues and in diseased tissues. *Journal of Infectious Diseases*.

[33] Monaco M. C. G., Atwood W. J., Gravell M., Tornatore C. S., Major E. O. (1996). JC virus infection of hematopoietic progenitor cells, primary B lymphocytes, and tonsillar stromal cells: Implications for viral latency. *Journal of Virology*.

[34] Power C., Gladden J. G. B., Halliday W. (2000). AIDS- and non-AIDS-related PML association with distinct p53 polymorphism. *Neurology*.

[35] Kappos L., Bates D., Edan G. (2011). Natalizumab treatment for multiple sclerosis: updated recommendations for patient selection and monitoring. *The Lancet Neurology*.

[36] US Food and Drug Administration Drug Safety and Availability—FDA Drug Safety Communication: FDA warns about cases of rare brain infection with MS drug Gilenya (fingolimod) in two patients with no prior exposure to immunosuppressant drugs. http://www.fda.gov/Drugs/DrugSafety/ucm456919.htm.

[37] US Food and Drug Administration Drug Safety and Availability—FDA Drug Safety Communication: FDA warns about case of rare brain infection PML with MS drug Tecfidera (dimethyl fumarate). http://www.fda.gov/Drugs/DrugSafety/ucm424625.htm.

[38] Van Oosten B. W., Killestein J., Barkhof F., Polman C. H., Wattjes M. P. (2013). PML in a patient treated with dimethyl fumarate from a compounding pharmacy. *The New England Journal of Medicine*.

[39] Ermis U., Weis J., Schulz J. B. (2013). PML in a patient treated with fumaric acid. *The New England Journal of Medicine*.

[40] Nieuwkamp D. J., Murk J., Cremers C. H. (2015). PML in a patient without severe lymphocytopenia receiving dimethyl fumarate. *The New England Journal of Medicine*.

[41] Langer-Gould A., Atlas S. W., Green A. J., Bollen A. W., Pelletier D. (2005). Progressive multifocal leukoencephalopathy in a patient treated with natalizumab. *The New England Journal of Medicine*.

[42] Kleinschmidt-DeMasters B. K., Tyler K. L. (2005). Progressive multifocal leukoencephalopathy complicating treatment with natalizumab and interferon beta-1a for multiple sclerosis. *The New England Journal of Medicine*.

[43] Van Assche G., Van Ranst M., Sciot R. (2005). Progressive multifocal leukoencephalopathy after natalizumab therapy for Crohn's disease. *The New England Journal of Medicine*.

[44] Yousry T. A., Major E. O., Ryschkewitsch C. (2006). Evaluation of patients treated with natalizumab for progressive multifocal leukoencephalopathy. *The New England Journal of Medicine*.

[45] Plavina T., Subramanyam M., Bloomgren G. (2014). Anti–JC virus antibody levels in serum or plasma further define risk of natalizumab-associated progressive multifocal leukoencephalopathy. *Annals of Neurology*.

[46] O'Connor P. (2012). Natalizumab risk stratification: role of a two- step anti-JCV antibody assay. *Canadian Journal of Neurological Sciences*.

[47] Dong-Si T., Richman S., Wattjes M. P. (2014). Outcome and survival of asymptomatic PML in natalizumab-treated MS patients. *Annals of Clinical and Translational Neurology*.

[48] Boster A., Hreha S., Berger J. R. (2009). Progressive multifocal leukoencephalopathy and relapsing-remitting multiple sclerosis: a comparative study. *Archives of Neurology*.

[49] Phan-Ba R., Lommers E., Tshibanda L. (2012). MRI preclinical detection and asymptomatic course of a progressive multifocal leucoencephalopathy (PML) under natalizumab therapy. *Journal of Neurology, Neurosurgery & Psychiatry*.

[50] Coyle P. K., Foley J. F., Fox E. J., Jeffery D. R., Munschauer F. E., Tornatore C. (2009). Best practice recommendations for the selection and management of patients with multiple sclerosis receiving natalizumab therapy. *Multiple Sclerosis*.

[51] Lima M. A., Drislane F. W., Koralnik I. J. (2006). Seizures and their outcome in progressive multifocal leukoencephalopathy. *Neurology*.

[52] Berger J. R., Aksamit A. J., Clifford D. B. (2013). PML diagnostic criteria: consensus statement from the AAN neuroinfectious disease section. *Neurology*.

[53] Mentzer D., Prestel J., Adams O. (2012). Case definition for progressive multifocal leukoencephalopathy following treatment with monoclonal antibodies. *Journal of Neurology, Neurosurgery and Psychiatry*.

[54] Phan-Ba R., Belachew S., Outteryck O. (2012). The earlier, the smaller, the better for natalizumab-associated PML: in MRI vigilance veritas?. *Neurology*.

[55] Weber T. (2008). Progressive multifocal leukoencephalopathy. *Neurologic Clinics*.

[56] Warnke C., Von Geldern G., Markwerth P. (2014). Cerebrospinal fluid JC virus antibody index for diagnosis of natalizumab-associated progressive multifocal leukoencephalopathy. *Annals of Neurology*.

[57] Kuhle J., Gosert R., Bühler R. (2011). Management and outcome of CSF-JC virus PCR-negative PML in a natalizumab-treated patient with MS. *Neurology*.

[58] Carroll B. A., Lane B., Norman D., Enzmann D. (1977). Diagnosis of progressive multifocal leukoencephalopathy by computed tomography. *Radiology*.

[59] Yousry T. A., Pelletier D., Cadavid D. (2012). Magnetic resonance imaging pattern in natalizumab-associated progressive multifocal leukoencephalopathy. *Annals of Neurology*.

[60] Piola M., Di Palma F., Mascoli N., Binda S., Arnaboldi M., Rezzonico M. (2014). Atypical MRI features at early onset natalizumab-associated progressive multifocal leukoencephalopathy: a case report. *Journal of the Neurological Sciences*.

[61] Gheuens S., Wüthrich C., Koralnik I. J. (2013). Progressive multifocal leukoencephalopathy: why gray and white matter. *Annual Review of Pathology: Mechanisms of Disease*.

[62] Koralnik I. J. (2006). Progressive multifocal leukoencephalopathy revisited: has the disease outgrown its name?. *Annals of Neurology*.

[63] Wattjes M. P., Barkhof F. (2014). Diagnosis of natalizumab-associated progressive multifocal leukoencephalopathy using MRI. *Current Opinion in Neurology*.

[64] Blair N. F., Brew B. J., Halpern J.-P. (2012). Natalizumab-associated PML identified in the presymptomatic phase using MRI surveillance. *Neurology*.

[65] Ayzenberg I., Lukas C., Trampe N., Gold R., Hellwig K. (2012). Value of MRI as a surrogate marker for PML in natalizumab long-term therapy. *Journal of Neurology*.

[66] Kleinschmidt-Demasters B. K., Miravalle A., Schowinsky J., Corboy J., Vollmer T. (2012). Update on PML and PML-IRIS occurring in multiple sclerosis patients treated with natalizumab. *Journal of Neuropathology and Experimental Neurology*.

[67] Wattjes M. P., Vennegoor A., Steenwijk M. D. (2015). MRI pattern in asymptomatic natalizumab-associated PML. *Journal of Neurology, Neurosurgery & Psychiatry*.

[68] Richert N., Bloomgren G., Cadavid D., Dong-Si T., Richman S., Ticho B. (2012). Imaging findings for PML in natalizumab-trated MS patients. *Multiple Sclerosis Journal*.

[69] Wattjes M. P., Richert N. D., Killestein J. (2013). The chameleon of neuroinflammation: magnetic resonance imaging characteristics of natalizumab-associated progressive multifocal leukoencephalopathy. *Multiple Sclerosis*.

[70] Kastrup O., Maschke M., Diener H. C., Wanke I. (2002). Progressive multifocal leukoencephalopathy limited to the brain stem. *Neuroradiology*.

[71] Mathew R. M., Murnane M. (2004). MRI in PML: bilateral medullary lesions. *Neurology*.

[72] Svensson P.-Å., Larsson E.-M. (2008). Infratentorial progressive multifocal leucoencephalopathy (PML) in a patient with SLE (2008: 4b). *European Radiology*.

[73] Semmler A., Urbach H., Klockgether T., Linnebank M. (2007). Progressive multifocal leukoencephalopathy with selective involvement of the pyramidal tracts. *Neurology*.

[74] Sarrazin J. L., Soulié D., Derosier C., Lescop J., Schill H., Cordoliani Y. S. (1995). MRI aspects of progressive multifocal leukoencephalopathy. *Journal of Neuroradiology*.

[75] Wattjes M. P., Verhoeff L., Zentjens W. (2013). Punctate lesion pattern suggestive of perivascular inflammation in acute natalizumab-associated progressive multifocal leukoencephalopathy: productive JC virus infection or preclinical PML-IRIS manifestation?. *Journal of Neurology, Neurosurgery and Psychiatry*.

[76] Tan I. L., McArthur J. C., Clifford D. B., Major E. O., Nath A. (2011). Immune reconstitution inflammatory syndrome in natalizumab-associated PML. *Neurology*.

[77] Roberts T. P. L., Mikulis D. (2007). Neuro MR: principles. *Journal of Magnetic Resonance Imaging*.

[78] Bergui M., Bradac G. B., Oguz K. K. (2004). Progressive multifocal leukoencephalopathy: diffusion-weighted imaging and pathological correlations. *Neuroradiology*.

[79] Küker W., Mader I., Nägele T. (2006). Progressive multifocal leukoencephalopathy: value of diffusion-weighted and contrast-enhanced magnetic resonance imaging for diagnosis and treatment control. *European Journal of Neurology*.

[80] Cosottini M., Tavarelli C., Del Bono L. (2008). Diffusion-weighted imaging in patients with progressive multifocal leukoencephalopathy. *European Radiology*.

[81] Mader I., Herrlinger U., Klose U., Schmidt F., Küker W. (2003). Progressive multifocal leukoencephalopathy: analysis of lesion development with diffusion-weighted MRI. *Neuroradiology*.

[82] Shah R., Bag A. K., Chapman P. R., Curé J. K. (2010). Imaging manifestations of progressive multifocal leukoencephalopathy. *Clinical Radiology*.

[83] Sahraian M. A., Radue E.-W., Eshaghi A., Besliu S., Minagar A. (2012). Progressive multifocal leukoencephalopathy: a review of the neuroimaging features and differential diagnosis. *European Journal of Neurology*.

[84] Usiskin S. I., Bainbridge A., Miller R. F., Jäger H. R. (2007). Progressive multifocal leukoencephalopathy: serial high-b-value diffusion-weighted MR imaging and apparent diffusion coefficient measurements to assess response to highly active antiretroviral therapy. *American Journal of Neuroradiology*.

[85] Da Pozzo S., Manara R., Tonello S., Carollo C. (2006). Conventional and diffusion-weighted MRI in progressive multifocal leukoencephalopathy: new elements for identification and follow-up. *Radiologia Medica*.

[86] Ohta K., Obara K., Sakauchi M., Obara K., Takane H., Yogo Y. (2001). Lesion extension detected by diffusion-weighted magnetic resonance imaging in progressive multifocal leukoencephalopathy. *Journal of Neurology*.

[87] Yoon J. H., Bang O. Y., Kim H. S. (2007). Progressive multifocal leukoencephalopathy in AIDS: proton MR spectroscopy patterns of asynchronous lesions confirmed by serial diffusion-weighted imaging and apparent diffusion coefficient mapping. *Journal of Clinical Neurology*.

[88] Chang L., Ernst T., Tornatore C. (1997). Metabolite abnormalities in progressive multifocal leukoencephalopathy by proton magnetic resonance spectroscopy. *Neurology*.

[89] Iranzo A., Moreno A., Pujol J. (1999). Proton magnetic resonance spectroscopy pattern of progressive multifocal leukoencephalopathy in AIDS. *Journal of Neurology, Neurosurgery & Psychiatry*.

[90] Fine A. J., Sorbello A., Kortepeter C., Scarazzini L. (2013). Central nervous system herpes simplex and varicella zoster virus infections in natalizumab-treated patients. *Clinical Infectious Diseases*.

[91] Yeung J., Cauquil C., Saliou G. (2013). Varicella-zoster virus acute myelitis in a patient with MS treated with natalizumab. *Neurology*.

[92] Kwiatkowski A., Gallois J., Bilbault N., Calais G., MacKowiak A., Hautecoeur P. (2012). Herpes encephalitis during natalizumab treatment in multiple sclerosis. *Multiple Sclerosis*.

[93] Eisele P., Szabo K., Griebe M. (2012). Reduced diffusion in a subset of acute MS lesions: a serial multiparametric MRI study. *American Journal of Neuroradiology*.

[94] Jander S., Turowski B., Kieseier B. C., Hartung H.-P. (2012). Emerging tumefactive multiple sclerosis after switching therapy from natalizumab to fingolimod. *Multiple Sclerosis*.

[95] Seewann A., Enzinger C., Filippi M. (2008). MRI characteristics of atypical idiopathic inflammatory demyelinating lesions of the brain: a review of reported findings. *Journal of Neurology*.

[96] Visser F., Wattjes M. P., Pouwels P. J. W., Linssen W. H. J. P., van Oosten B. W. (2012). Tumefactive multiple sclerosis lesions under fingolimod treatment. *Neurology*.

[97] Tenembaum S., Chitnis T., Ness J., Hahn J. S. (2007). Acute disseminated encephalomyelitis. *Neurology*.

[98] Lamy C., Oppenheim C., Méder J. F., Mas J. L. (2004). Neuroimaging in posterior reversible encephalopathy syndrome. *Journal of Neuroimaging*.

[99] Khatri B. O., Man S., Giovannoni G. (2009). Effect of plasma exchange in accelerating natalizumab clearance and restoring leukocyte function. *Neurology*.

[100] Vennegoor A., Rispens T., Van Oosten B. W. (2014). Application of serum natalizumab levels during plasma exchange in MS patients with progressive multifocal leukoencephalopathy. *Multiple Sclerosis Journal*.

[101] Shelburne S. A., Hamill R. J., Rodriguez-Barradas M. C. (2002). Immune reconstitution inflammatory syndrome: emergence of a unique syndrome during highly active antiretroviral therapy. *Medicine*.

[102] Wattjes M. P., Killestein J. (2014). Progressive multifocal leukoencephalopathy after natalizumab discontinuation: few and true?. *Annals of Neurology*.

[103] Salhofer-Polanyi S., Baumgartner A., Kraus J., Maida E., Schmied M., Leutmezer F. (2014). What to expect after natalizumab cessation in a real-life setting. *Acta Neurologica Scandinavica*.

[104] West T. W., Cree B. A. C. (2010). Natalizumab dosage suspension: are we helping or hurting?. *Annals of Neurology*.

[105] West T. W., Killestein J., Fox R. J. (2012). Natalizumab discontinuation: an increasingly tricky proposition. *European Journal of Neurology*.

[106] Kerbrat A., Le Page E., Leray E. (2011). Natalizumab and drug holiday in clinical practice: an observational study in very active relapsing remitting Multiple Sclerosis patients. *Journal of the Neurological Sciences*.

[107] Rigau V., Mania A., Befort P. (2012). Lethal multiple sclerosis relapse after natalizumab withdrawal. *Neurology*.

[108] Sorensen P. S., Koch-Henriksen N., Petersen T., Ravnborg M., Oturai A., Sellebjerg F. (2014). Recurrence or rebound of clinical relapses after discontinuation of natalizumab therapy in highly active MS patients. *Journal of Neurology*.

[109] Miravalle A., Jensen R., Kinkel R. P. (2011). Immune reconstitution inflammatory syndrome in patients with multiple sclerosis following cessation of natalizumab therapy. *Archives of Neurology*.

[110] Beume L.-A., Dersch R., Fuhrer H., Stich O., Rauer S., Niesen W. D. (2015). Massive exacerbation of multiple sclerosis after withdrawal and early restart of treatment with natalizumab. *Journal of Clinical Neuroscience*.

[111] Vellinga M. M., Castelijns J. A., Barkhof F., Uitdehaag B. M. J., Polman C. H. (2008). Postwithdrawal rebound increase in T2 lesional activity in natalizumab-treated MS patients. *Neurology*.

[112] Sangalli F., Moiola L., Ferrè L. (2014). Long-term management of natalizumab discontinuation in a large monocentric cohort of multiple sclerosis patients. *Multiple Sclerosis and Related Disorders*.

[113] O'Connor P. W., Goodman A., Kappos L. (2011). Disease activity return during natalizumab treatment interruption in patients with multiple sclerosis. *Neurology*.

[114] Jokubaitis V. G., Li V., Kalincik T. (2014). Fingolimod after natalizumab and the risk of short-term relapse. *Neurology*.

[115] Fox R. J., Cree B. A. C., De Séze J. (2014). MS disease activity in RESTORE: a randomized 24-week natalizumab treatment interruption study. *Neurology*.

[116] Chaves C., Ganguly R., Dionne C., Camac A. (2015). Relapse and rebound risks after natalizumab discontinuation in patients with multiple sclerosis. (P3.294). *Neurology*.

[117] Clares R. H., Torres R. C., Carreon E., Andreu E., La Llana J. M., Fernandez M. (2015). Risk of rebound in multiple sclerosis after a switch from natalizumab to fingolimod. Early onset versus delayed onset. (P3.291). *Neurology*.

